# DHX9 helicase promotes R-loop formation in cells with impaired RNA splicing

**DOI:** 10.1038/s41467-018-06677-1

**Published:** 2018-10-19

**Authors:** Prasun Chakraborty, Jeffrey T. J. Huang, Kevin Hiom

**Affiliations:** 1 Division of Cellular Medicine, School of Medicine, University of Dundee, Scotland, UK; 2 Biomarker and Drug Analysis Core Facility, School of Medicine, University of Dundee, Scotland, UK

## Abstract

R-loops are stable nucleic acid structures that have important physiological functions, but which also pose a significant threat to genomic stability. Increased R-loops cause replication stress and chromosome fragility and have been associated with diseases such as neurodegeneration and cancer. Although excessive R-loops are a feature of cells that are defective in RNA processing, what causes them to form is unclear. Here, we demonstrate that DHX9 (RNA helicase A) promotes the formation of pathological and non-pathological R-loops. In the absence of splicing factors, formation of R-loops correlates with the prolonged association of DHX9 with RNA Polymerase II (RNA Pol II). This leads to the production of DNA–RNA hybrid, which traps RNA Pol II on chromatin with the potential to block DNA replication. Our data provide a molecular mechanism for the formation of R-loops that is relevant to neurodegenerative diseases and cancers in which deregulated RNA processing is a feature.

## Abstract (web summary)

Unresolved R-loops can represent a threat to genome stability. Here the authors reveal that DHX9 helicase can promote R-loop formation in the absence of splicing factors SFPQ and SF3B3.

## Introduction

R-loops are generated during transcription when nascent RNA exits RNA polymerase and pairs with its complementary DNA template to form a region of RNA–DNA hybrid and displaced single-stranded DNA (ssDNA)^1^. R-loops are found in a broad range of organisms where they function in a variety of cellular processes, including replication of mitochondrial genomes and bacterial plasmids, regulation of chromosome segregation^2^, and immunoglobulin class-switch recombination^3^. In mammalian cells R-loops are widespread, occupying as much as 5% of the genome and are enriched at promoter and terminator regions of polyA-dependent genes, suggesting that they might play a role in the regulation of gene expression^4,5^. R-loops are also found in rDNA and tRNA genes suggesting that they form during transcription involving RNA Polymerases I, II, or III^6^.

However, R-loops can pose a significant threat to genomic stability in a variety of ways^7,8^. Firstly, the displaced single-stranded DNA in R-loops is vulnerable to attack from the APOBEC family of cytosine deaminases which, upon further processing by enzymes of the base excision repair pathway, may lead to the generation of single-stranded DNA breaks^9^. Secondly, regions of transition from single-strand DNA to double-stranded DNA at the extremities of R-loops can be cleaved by proteins of the nucleotide excision repair pathway, generating double-stranded DNA breaks (dsb)^10^. Lastly, by impeding the progression of RNA polymerase on DNA, R-loops increase the potential for transcription–replication conflicts (TRC)^11–14^. This can lead to stalling and collapse of replication forks and the production of one-ended dsb that are substrates for chromosome translocations^6,15,16^. In humans, increased R-loops are found in a variety of diseases that exhibit genomic instability, including myelodysplastic syndromes^17^, neurodegenerative diseases^18,19^, and cancers such as Ewing’s sarcoma^20^.

Given the potential of R-loops to cause genomic instability, the accumulation of these structures in cells must be tightly regulated. Indeed, a variety of proteins have been identified that prevent R-loops from forming. The majority of these are proteins involved in ribonucleoprotein (RNP) biogenesis and pre-mRNA processing, including several splicing factors and components of the THO/TREX complex that couples the maturation and export of pre-mRNA^21,22^. In both yeast and human cells, defects in these proteins leads to the accumulation of R-loops and increased DNA damage.

Several other proteins facilitate the removal of R-loops. RNaseH1, for example, removes R-loops by specifically degrading RNA–DNA hybrid^23^. Alternatively, helicases including SETX (Sen1 in yeast) and AQR, disassemble R-loops by unwinding RNA–DNA hybrid^24–26^. Interestingly, the DNA repair protein BRCA2 also suppresses R-loops by promoting release of RNA Pol II that is paused at a promoter region^27,28^. However, it is unclear how these different factors regulate the balance between formation and removal of R-loops to prevent the pathological potential of these stable nucleic acid structures in cells.

Although R-loops have been shown to play specific roles in normal physiological processes and to accumulate in cells that are defective in RNA metabolism, it is still unclear what causes R-loops to form and whether this requires the activities of specific proteins. We investigated the role of splicing factors in R-loop-induced replication stress and identified the RNA helicase, DHX9, as a key factor in the generation of R-loops by RNA Polymerase II. Our data shed new light on the mechanism through which R-loops are formed and the important role played by splicing factors to prevent R-loop induced replication stress and genomic instability.

## Results

### Defects in SFPQ cause R-loop induced DNA replication stress

An increasing number of proteins that function in RNA metabolism have also been shown to contribute to the maintenance of genomic stability^29^. Among these are members of the Drosophila Behavior and Human Splicing (DBHS) family of proteins, which are found in subnuclear bodies called paraspeckles^30^. Although DBHS proteins are required for the retention and processing of hyper-edited RNAs, some also play a role in the repair of dsb by homologous recombination and non-homologous end-joining^31,32^. We focused on one of these, SFPQ (splicing factor proline and glutamine rich), and found that it promotes genomic stability by preventing the formation of R-loops.

**Fig. 1:**
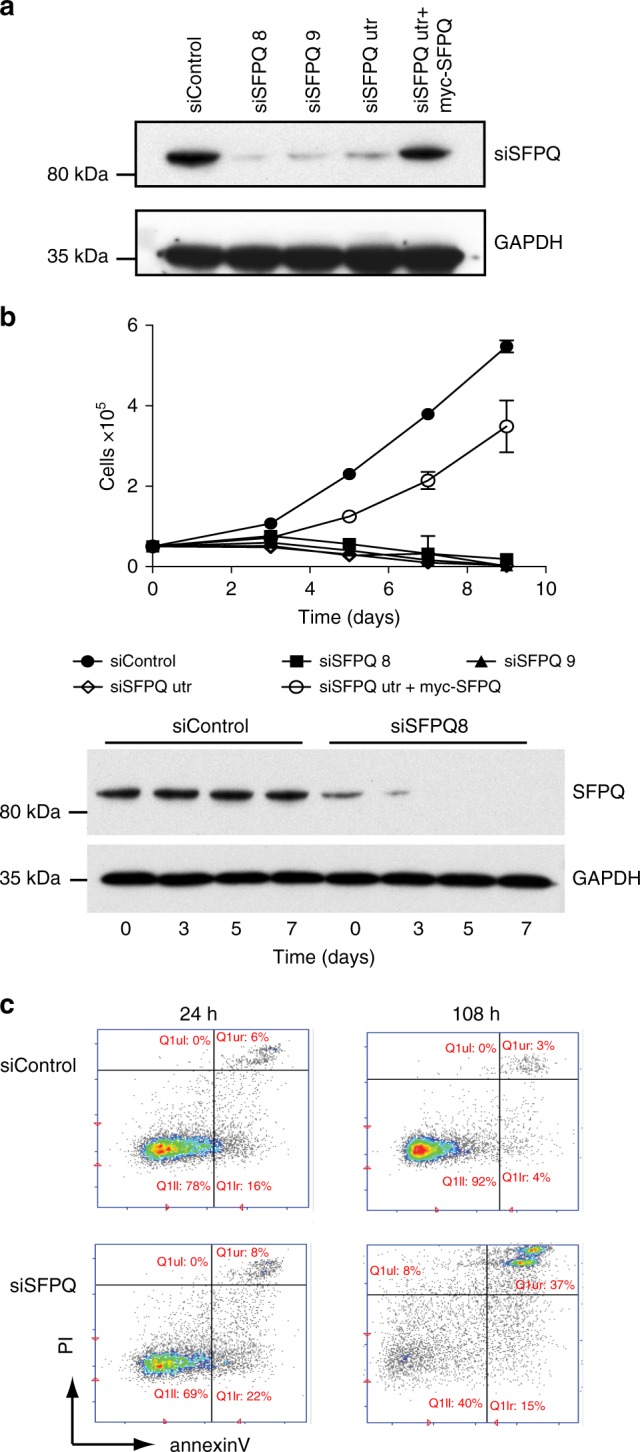
Depletion of SFPQ impairs cell growth. **a** Western blot showing knockdown of SFPQ using different siRNAs. Expression of myc-SFPQ is resistant to knockdown by siSFPQutr. **b** Cell proliferation in U2OS cells transfected with different siRNAs targeting SFPQ and a non-specific sequence (siControl). Cell number was measured 48 h after transfection with siRNA (time = 0) and then at time = 3, 5, 7, and 9 days. Overexpression of siRNA-resistant myc-SFPQ rescued cell growth in SFPQ knockdown cells. Data are an average *n* = 3 independent experiments ± s.d. Western blot (below) shows the level SFPQ protein over time. **c** Knockdown of SFPQ (siSFPQ9) leads to increased apoptosis, quantified by staining for Annexin-V

**Fig. 2:**
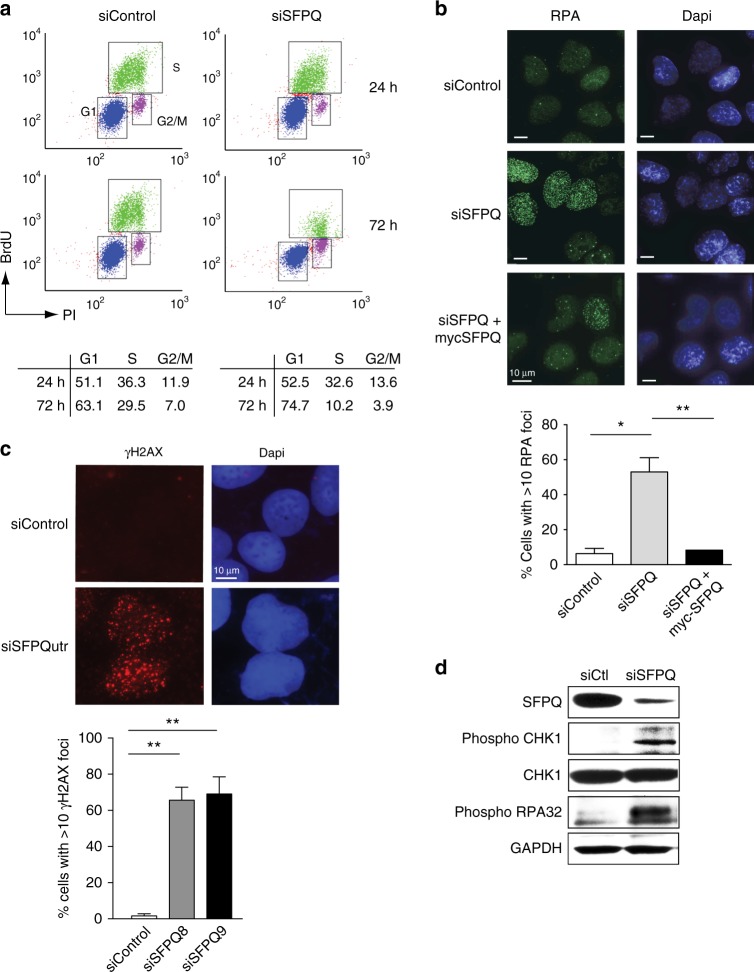
Defects in SFPQ cause replication stress. **a** FACS analysis showing that knockdown of SFPQ (siSFPQ9) leads to a reduction in the s-phase cells. Quantification of FACS data was averaged from three independent experiments. **b** Microscope images showing that knockdown of SFPQ with siSFPQ8 in U2OS cells leads to increased RPA foci. Expression of exogenous myc-SFPQ (indicated) resulted in partial suppression of RPA foci in SFPQ-depleted cells. Quantification is shown below and indicate mean percentage of cells with >10 RPA foci ± s.d. More than 100 cells were counted from each of three independent experiments. Statistical significance was determined using Student’s *t*-test (*<0.05 and **<0.01). **c** Images showing increased γH2AX foci in U2OS cells knocked down with siSFPQutr compared with control cells. Graph shows mean percentage of cells with >5 γH2AX foci ± s.d. Data are plotted from three independent experiments. Statistical significance was determined using Student’s *t*-test (**p < 0.01). **d** Western blot showing that knockdown of SFPQ with siSFPQ8 leads to the phosphorylation of CHK1 on serine 345 and phosphorylation of RPA32 on Ser4/Ser8

**Fig. 3:**
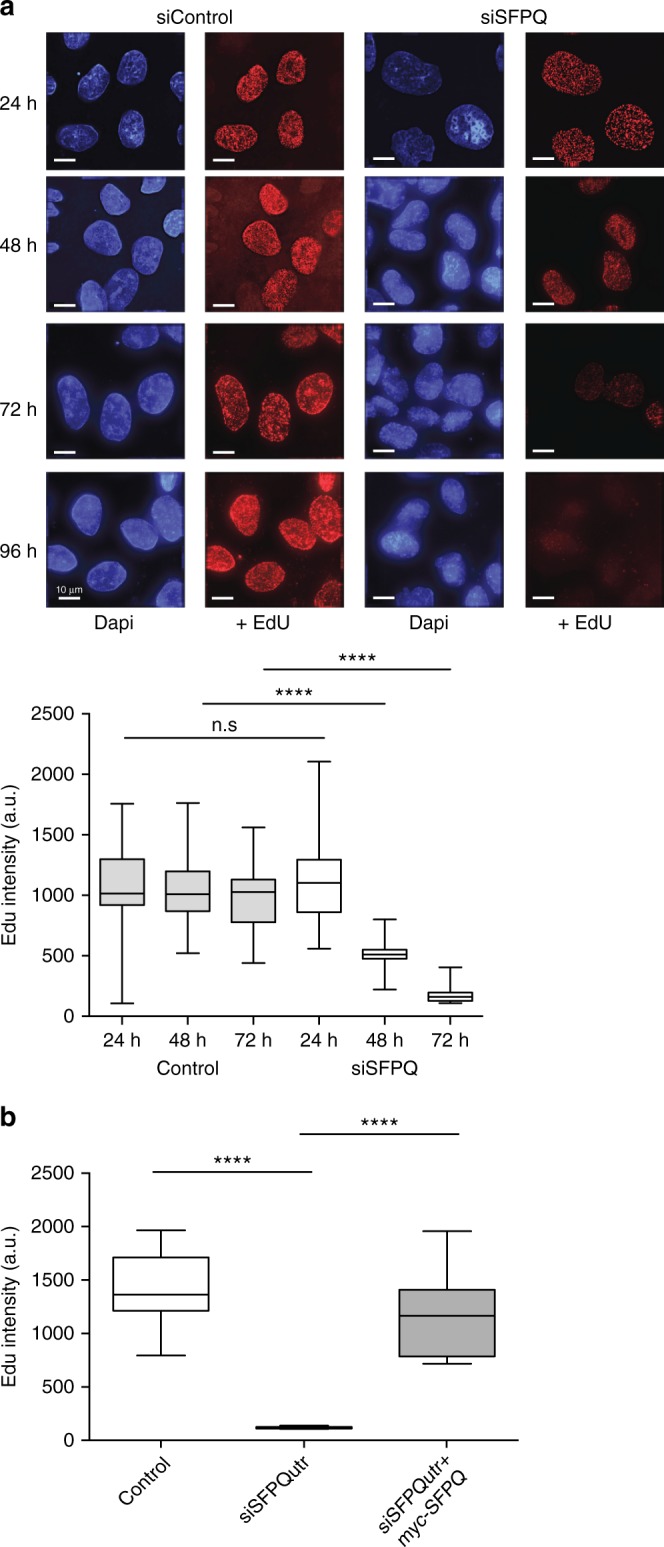
Defects in SFPQ cause impaired DNA synthesis. **a** Microscope images showing that, in U2OS cells, incorporation of EdU into DNA over time is diminished by knockdown of SFPQ (siSFPQ8). Immunofluorescence intensity of nuclear EdU was quantified from >100 cells and is presented graphically as a box and whiskers plot. Statistical significance was determined using Mann–Whitney test (****p < 0.0001). Scale bars represent 10 μm. **b** Expression of siRNA-resistant myc-SFPQ in U2OS cells restores DNA synthesis. EdU fluorescence intensity from >100 cells is depicted in arbitrary units (a.u.). Statistical significance was determined using Mann–Whitney test (****p < 0.0001). Box and whisker plots display median, upper and lower quartile range with whiskers depicting lowest and highest values

Homozygous deletion of *Sfpq* in mice is embryonically lethal^33^. Accordingly siRNA-mediated knockdown of SFPQ in U2OS cells (Fig. 1a) led to impaired cell growth (Fig. 1b) and increased apoptotic cell death (Fig. 1c), confirming that it is essential for cell viability. Importantly, viability was restored by exogenous expression of an siRNA-resistant SFPQ-myc gene in these cells (Fig. 1b). Several pieces of evidence indicated that this defect in cell proliferation was caused by impaired DNA synthesis. Firstly, knockdown of SFPQ led to a reduction in the number of S-phase cells compared to the control populations (Fig. 2a). Secondly, depletion of SFPQ induced severe replication stress, characterized by the production of excessive single-stranded DNA (RPA foci) (Fig. 2b), increased DNA double-stranded breaks (γH2AX foci) (Fig. 2c), and phosphorylation of the single-strand binding protein RPA32 (Fig. 2d). Thirdly, we observed activation of the replication checkpoint as indicated by phosphorylation of Chk1, a substrate of the ATR checkpoint kinase (Fig. 2d). Lastly, SFPQ-depleted cells were unable to efficiently incorporate the nucleotide analog EdU (5-ethynyl-2′-deoxyuridine) into DNA (Fig. 3a), indicating a failure in DNA synthesis. Importantly, replication stress (Fig. 2b) and DNA synthesis (Fig. 3b) were rescued by over-expressing siRNA-resistant SFPQ-myc cDNA in cells.

Although SFPQ plays a role in RNA processing, several pieces of evidence argued that the replication stress, which accompanied knockdown of SFPQ, was not caused by impaired expression of proteins required in DNA synthesis. Firstly, treatment of wild-type cells with the transcription inhibitor actinomycin D did not generate the increased RPA foci that were evident in SFPQ-defective cells (Supplementary Fig. 1a), nor did it impair DNA synthesis (Supplementary Fig. 1b). Secondly, actinomycin D treatment diminished RPA staining (Supplementary Fig. 1a) and partially restored DNA replication (Supplementary Fig. 1a) in SFPQ-depleted cells, indicating that replication stress caused by defects in SFPQ requires active transcription. Lastly, replication stress in SFPQ-depleted cells closely resembled that caused by hydroxyurea, a potent inhibitor of DNA synthesis, which causes replication forks to stall and collapse (Supplementary Fig. 1a).

**Fig. 4:**
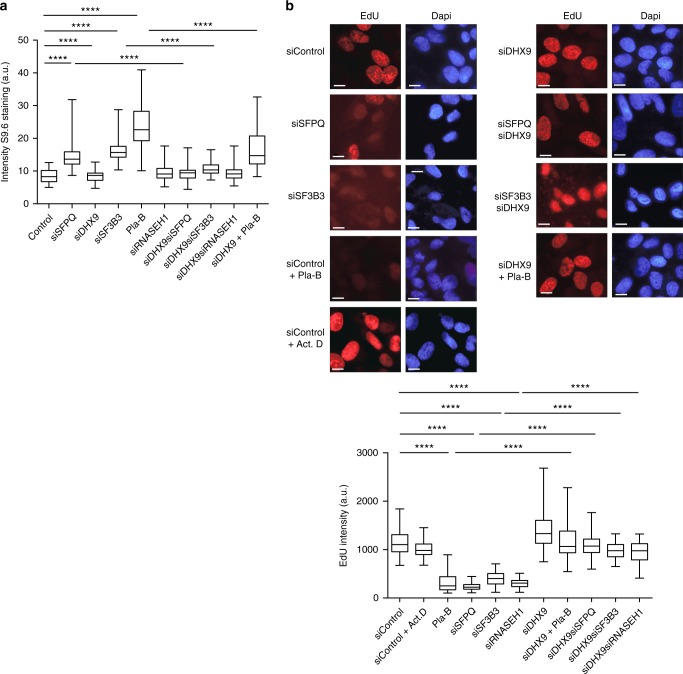
Defects in RNA splicing cause increased R-loops. **a** Inhibition of RNA splicing leads to elevated levels of RNA–DNA hybrids that are suppressed by knockdown of DHX9. Cells were transfected with siRNAs targeted to the indicated genes, individually and in combination. Where indicated, cells were treated with Pla-B (5μM) for 2 h to inhibit splicing. **b** Incorporation of EdU into DNA 72 h after knockdown of the indicated genes. Where indicated, cells were treated with Pla-B (5 μm) or Actinomycin D (0.5 μg/ml). All scale bars represent 10 μm. Graphical data from *n* > 90 cells are presented. Statistical significance for all graphs was determined using Mann–Whitney test (****p < 0.0001)

Intriguingly, SFPQ is also a component of the spliceosome C complex that is required for the formation of RNA lariats during pre-mRNA splicing^31^. Defects in RNA splicing factors, such as ASF/SRSF1, lead to increased genomic instability resulting from the formation of R-loops^22^. Therefore, we next investigated whether the replication stress and impaired cell growth in SFPQ-depleted cells was also linked to the generation of R-loops. To do this, we used S9.6 antibody^34^ to detect and quantify the RNA–DNA hybrid component of R-loops. Using S9.6 in fluorescence imaging (Supplementary Fig. 2a) and slot blot hybridization (Supplementary Fig. 2b), we observed that loss of SFPQ was accompanied by a significant increase in RNA–DNA hybrids that was reversed by expressing siRNA-resistant SFPQ-myc (Supplementary Fig. 2c). This was also true in cells treated with the splicing inhibitor Pladeinolide B (Pla-B) and in cells knocked down for splicing factor SF3B3, the protein targeted by Pla-B^35^(Fig. 4a). In both cases RNA–DNA hybrids were increased, leading to impaired DNA synthesis (Fig. 4b). Importantly, R-loops were diminished upon the transient expression of RNaseH1 (Supplementary Fig. 2d), which degrades RNA–DNA hybrids, or by treating cells with the transcription inhibitor actinomycin D (Supplementary Fig. 2c). These data confirm the importance of the splicing machinery generally and SFPQ, specifically, in preventing the formation of R-loops and promoting genomic stability in cells.

### R-loop formation requires DHX9 helicase

**Fig. 5:**
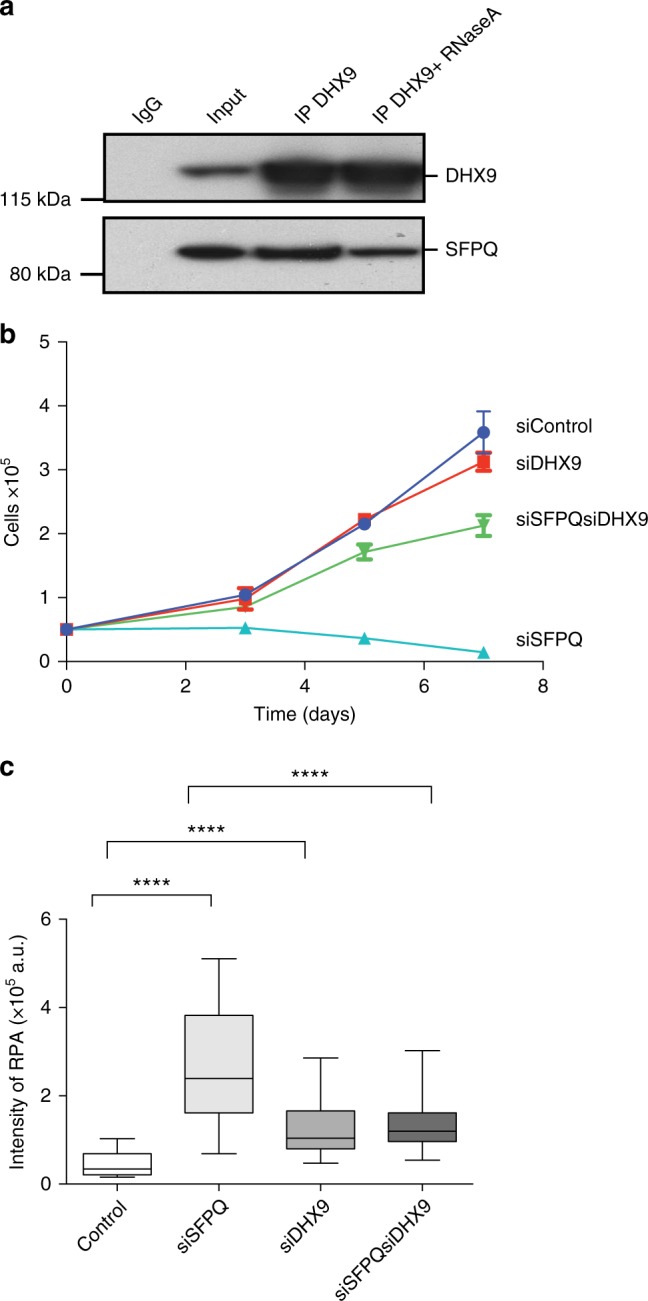
DHX9 interacts with SFPQ. **a** Western blot showing co-immunoprecipitation of SFPQ with DHX9 from Hela cell nuclear extract. Control sample treated with RNaseA is indicated. Input sample and IgG control are also shown. **b** Knockdown of DHX9 suppresses the growth defect in SFPQ-depleted cells (siSFPQ8). Cell number was measured 48 h after transfection with siRNA (time 0) and again after 3, 5, and 7 days. **c** Knockdown of DHX9 suppresses replication stress in SFPQ-depleted cells. Fluorescence intensity of RPA foci was measured in *n* > 90 cells, 72 h after transfection with the indicated siRNA. Statistical significance was determined using Mann–Whitney test (****p < 0.0001). Box and whisker plots display median, upper and lower quartile range with whiskers depicting lowest and highest values

An important question arising from our data was how do RNA processing factors, such as SFPQ, prevent R-loops from forming? Recently, we and others found that SFPQ co-immunoprecipitated from nuclear cell extracts with the DExH-type helicase DHX9, as detected by western blot (Fig. 5a)^36^ and confirmed by mass spectrometry (Supplementary Fig. 3). Although both proteins bind RNA, the interaction between DHX9 and SFPQ was not mediated by RNA as it was resistant to treatment with RNase A (Fig. 5a).

Although DHX9 has been implicated in many fundamental cellular processes including DNA replication, transcription, and genome stability, its specific biological function remains unclear^37^. Nevertheless, we were intrigued by the fact that DHX9 can unwind short (seventeen nucleotide) RNA–DNA hybrids in vitro^38^ and hypothesized that if it also unwinds RNA–DNA hybrid in vivo, knockdown of DHX9 should lead to increased R-loop formation. However, when DHX9 was depleted in U2OS cells, we observed no increase in R-loops (Fig. 4a, Supplementary Fig. 2b) and both DNA replication (Fig. 4b) and cell growth (Fig. 5b) were normal. Therefore, unlike SFPQ, DHX9 did not contribute to the prevention of R-loops.

However, when SFPQ and DHX9 were knocked down simultaneously, R-loops were suppressed (Fig. 4a) and replication stress (Fig. 5c) was alleviated compared to cells defective in SFPQ alone. Moreover, DNA synthesis (Fig. 4b) and cell proliferation (Fig. 5b) were restored to near wild-type levels in these cells. This indicated that in cells lacking SFPQ, DHX9 promotes the formation of R-loops. Importantly, the contribution of DHX9 to the production of R-loops and associated replication stress was not limited to cells defective in SFPQ. Depletion of DHX9 also suppressed R-loops (Fig. 4a) and restored DNA replication (Fig. 4b) in cells treated with Pla-B or depleted of SF3B3. This led us to two important conclusions. Firstly, in cells defective in RNA splicing the formation of R-loops is dependent on DHX9. Secondly, that by promoting the formation of R-loops, DHX9 contributes to the pathological replication stress caused by perturbations in RNA splicing.

### R-loops form along the coding region of the beta-actin gene

**Fig. 6:**
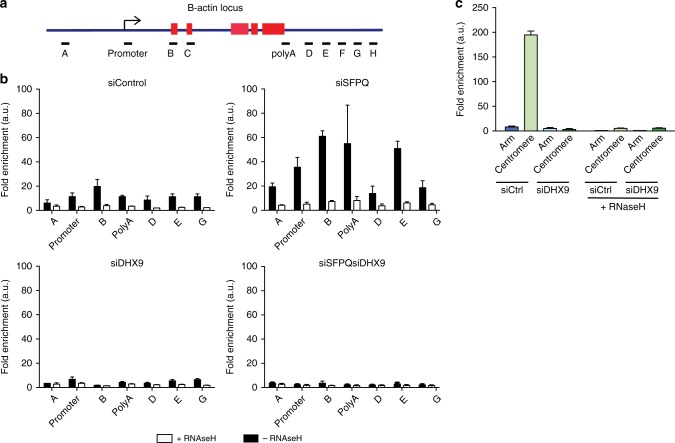
DHX9 promotes the formation of pathological and non-pathological R-loops. **a** Diagram of the *Β-actin* locus depicting relative position of primer pairs used for qPCR in DNA–RNA immunoprecipitation (DRIP) and chromatin immunoprecipitation (ChIp) experiments. Exons are depicted as red boxes. **b** DRIP analysis at the *Β-actin* locus using S9.6 antibody. Quantitative PCR was performed using primers to the indicated regions of the *Β-actin* locus. Data are represented as fold enrichment compared to control immunoprecipitations. Data are an average of three independent experiments. **c** DRIP analysis of RNA–DNA hybrid at centromeric and flanking arm regions of chromosome 1 in cells knocked down for DHX9. Samples were treated with RNaseH1 to remove DNA–RNA hybrid are indicated

To determine how R-loops form at a specific gene, we used S9.6 antibody for DNA–RNA immunoprecipitation (DRIP) analysis at the *Β-actin* locus (Fig. 6a). This confirmed that knockdown of SFPQ causes a significant increase in RNA–DNA hybrids compared to control cells (Fig. 6b). Moreover, it revealed that RNA–DNA hybrids can form along the entire length of the gene coding region, beginning upstream of the *Β-actin* promoter, extending through the gene body and terminating downstream of the polyA site (Fig. 6b). Hence, the formation of RNA–DNA hybrids probably begins early in transcription and continues throughout elongation until transcription is terminated. Interestingly, we detected a second peak of RNA–DNA hybrids downstream from the polyadenylation site (region E) (Fig. 6b), which might reflect transcripts that have undergone 3′end-processing. Importantly, knockdown of DHX9 suppressed the formation of RNA–DNA hybrids across the whole coding region, confirming our hypothesis that the generation of R-loops in SFPQ-defective cells is dependent on DHX9.

We also detected a basal level of RNA–DNA hybrids at the *Β-actin* locus in control cells. These too were diminished after knockdown of DHX9 (Fig. 6b), suggesting that DHX9 is also involved in mechanisms leading to the low-level R-loops formed during transcription in normal cells.

### DHX9 promotes the formation of non-pathological R-loops

Previous studies have shown that knockdown of XRN2, an exoribonuclease required in 3′ processing of mRNA, also leads to an accumulation of R-loops. Intriguingly, the recruitment of XRN2 to nascent RNA is dependent on a complex comprising SFPQ and p54^nrb^, raising the possibility that SFPQ and XRN2 function together to prevent R-loop formation^39^. However, we found, in contrast with a previous study^40^, that depletion of XRN2 induced the generation of R-loops (Supplementary Fig. 4a), particularly at the 3′ coding and termination regions (Supplementary Fig. 4b), but did not inhibit DNA synthesis (Supplementary Fig. 4c), nor did it impair cell growth (Supplementary Fig. 4d). In fact, incorporation of EdU into DNA was enhanced in cells knocked down for XRN2, compared with control cells (Supplementary Fig. 4c). This suggested that the contribution of SFPQ to R-loop formation must be independent of its physical association with XRN2. Moreover, it showed that the R-loops formed in cells knocked down for XRN2 did not cause pathological effects on DNA replication. Interestingly, these R-loops were also suppressed by knockdown of DHX9 (Supplementary Fig. 4a).

Although perturbations in RNA processing often lead to R-loops that are deleterious for DNA replication and cell growth, R-loops that play a role in normal cell processes are, presumably, not harmful. It was reported recently that R-loops found at chromosome centromeres cause the local activation of ATR, which regulates phosphorylation of the mitotic regulator protein Aurora B during chromosome segregation^2^. To determine if DHX9 was also required in the formation of these R-loops we performed DRIP on centromeric DNA and DNA in the flanking arms of chromosome 1. As expected, RNA–DNA hybrid was enriched at the centromeric DNA of chromosome 1, but not in its flanking arms (Fig. 6c). Critically, knockdown of DHX9 suppressed centromeric RNA–DNA hybrid, confirming that these naturally occurring R-loops are also generated through a mechanism involving DHX9.

### Requirement for the ATP-dependent helicase function of DHX9

**Fig. 7:**
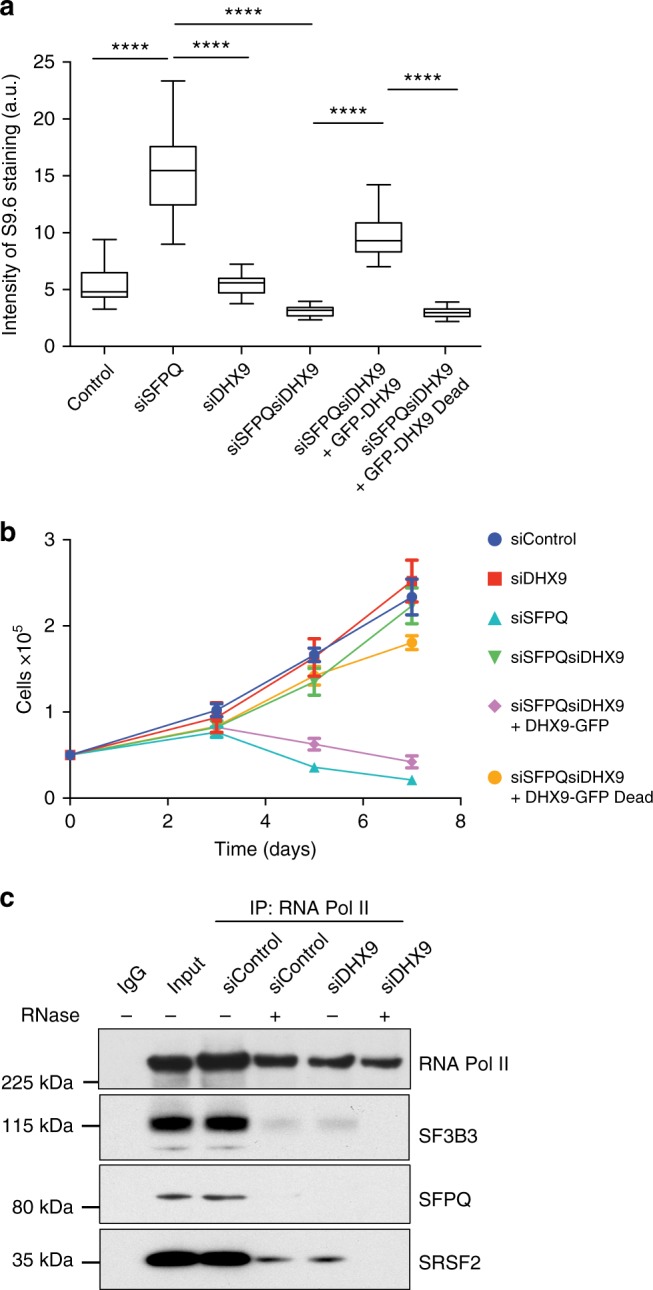
DHX9 helicase activity is required for R-loop formation and growth inhibition. **a** Expression of wild-type DHX9, but not a helicase-defective mutant (pGFP-DHX9dead), promotes R-loop formation in SFPQ-defective cells. Fluorescence intensity of S9.6 staining was measured for *n* > 50 cells. Statistical significance was determined using Mann–Whitney test (****p < 0.0001). **b** Expression of wild-type DHX9 but not a helicase-defective mutant confers impaired cell proliferation in SFPQ-depleted cells (siSFPQ8). Cell number was measured using a Casey Cell Counter 48 h after transfection with siRNA (time 0) and again after 3, 5, and 7 days. **c** RNA Pol II S2P was immunoprecipitated from Hela cell nuclear extracts and co-purification of different splicing factors was probed by western blot (as indicated). Duplicate samples were treated with RNaseA as indicated to demonstrate the requirement of RNA in these interactions

We next addressed the mechanism through which DHX9 promotes R-loop formation. In vitro, DHX9 unwinds a variety of nucleic acid structures in reactions that are dependent on ATP hydrolysis^38,41^. To determine if the helicase activity of DHX9 is required in cells to generate R-loops, we transfected an siRNA-resistant-GFP-tagged wild-type DHX9 (GFP-DHX9) and a helicase/ATPase-dead mutant of DHX9 (GFP-DHX9dead) into cells knocked down for the endogenous DHX9 and SFPQ genes. Whereas expression of GFP-DHX9 rescued R-loop formation in siSFPQsiDHX9 double-knockdown cells (Fig. 7a), leading to inhibition of DNA replication and impaired cell growth (Fig. 7b), expression of the GFP-DHX9dead mutant did not (Fig. 7). This confirmed that the ATP-dependent unwinding activity of DHX9 is essential for the generation of R-loops in SFPQ-depleted cells.

### DHX9 promotes binding of splicing factors to nascent RNA

DHX9 unwinds short RNA–DNA hybrids in vitro, suggesting that it might prevent R-loops from forming in cells^38^. However, our data established that DHX9 is required for the formation of R-loops in cells and, therefore, that RNA–DNA hybrid is unlikely to be the physiological substrate for DHX9 unwinding activity. However, DHX9 also unwinds quadruplex RNA in vitro^38,41^ and in cells has been shown to promote splicing of the GluR-B gene by unwinding an inhibitory RNA stem–loop, suggesting that its physiological substrate is most likely RNA secondary structures^42^. We speculated that DHX9 might promote R-loop formation by unwinding the nascent RNA strand to generate a free RNA end that can invade duplex DNA to form RNA–DNA hybrid^43^. We further hypothesized that in normal cells this activity might facilitate the binding of RNA processing proteins, such as splicing factors to nascent RNA.

To test this, we immunoprecipitated RNA Pol II from wild-type cells that were depleted of DHX9, and looked for co-purification of splicing factors SFPQ, SRSF1, and SF3B3 (Fig. 7c). In normal cells we found that all three splicing factors co-precipitated with RNA Pol II. We confirmed that these interactions were mediated through RNA by showing that they were diminished in samples treated with RNaseA. However, in cells knocked down for DHX9, co-purification of SFPQ, SRSF1, and SF3B3 with RNA Pol II was greatly reduced, supporting our hypothesis that DHX9 facilitates the assembly of splicing factors on nascent RNA during transcription.

### Prolonged interaction of DHX9 with the RNA Pol II

In normal cells, DHX9 helicase activity promotes the assembly of splicing factors onto nascent RNA and, in the absence of splicing factors, it promotes the formation of R-loops. These data led us to hypothesize that the assembly of splicing factors on nascent RNA strand might be a critical event that prevents R-loops from forming. Therefore, we investigated whether the availability of splicing factors affected DHX9 function during transcription and how this might be related to the generation of R-loops.

In cells, binding of splicing factors to pre-mRNA and the removal of introns occurs concurrently with transcription and is coordinated through interactions between the splicing machinery and the carboxy-terminal domain (CTD) of RNA Pol II^44^. These interactions are regulated through the phosphorylation of the CTD on different serine residues at different stages of transcription^45^. RNA Pol II that is phosphorylated on serine 5 of the CTD (S5P) is enriched at promoter regions and diminishes towards the 3′ end of the genes, while serine 7 phosphorylation (S7P) remains high throughout transcription. On the other hand, phosphorylation on serine 2 of the CTD (S2P) is low at promoter regions and increases progressively toward the 3′ end of genes^44^.

**Fig. 8:**
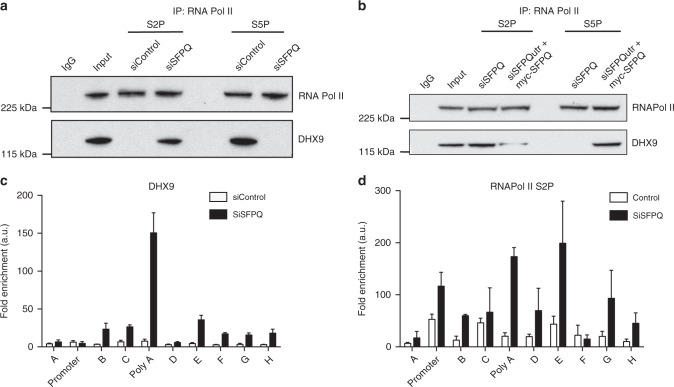
DHX9 associates with different phosphorylated forms of RNA Polymerase II. **a** Western blot of RNA Pol II and DHX9 immunoprecipitated from HeLa cells with antibodies specific for phosphor-serine 2 (S2P) and phosphor-serine 5 (S5P) forms of RNA Pol II. Where indicated cells were transfected with siRNA against a scrambled DNA sequence (siControl) or against SFPQ (siSFPQ8, siSFPQ9). **b** Expression of siRNA-resistant myc-SFPQ in SFPQ knockdown cells promotes the association of DHX9 with RNA Pol II S5P and diminishes its association with S2P. **c** Chromatin immunoprecipitation of DHX9 at the *Β-actin* locus. Data are depicted as fold enrichment over the control IP. qPCR of IP samples was performed for the primer pairs described in Fig. 6a. Means and s.e.m. are plotted and data are an average from three independent replicates. **d** Chromatin immunoprecipitation of RNA Pol II S2P at the *Β-actin* locus as described in **c**

We immunoprecipitated different phosphorylated forms of RNA Pol II and looked for co-precipitation of DHX9. In control cells, DHX9 co-purified primarily with S5P (Fig. 8a) and to a small extent with S7P (Supplementary Fig. 5a), but not with S2P (Fig. 8a). This indicated that DHX9 associated with RNA Pol II early in transcription, but dissociated at some point during elongation. In cells depleted for either SFPQ (Fig. 8a) or SF3B3 (Supplementary Fig. 5b), DHX9 co-precipitated primarily with S2P but not with S5P, implying that perturbation of RNA splicing caused DHX9 to remain associated with the transcription complex throughout elongation until termination. Importantly, exogenous expression of a siRNA-resistant form of SFPQ re-established the association of DHX9 with S5P and diminished its association with S2P (Fig. 8b).

Chromatin immunoprecipitation (ChIP) of DHX9 and RNA Pol II at the *Β-actin* locus confirmed that, in the absence of SFPQ, both DHX9 (Fig. 8c) and RNA Pol II (Fig. 8d) were highly enriched at the polyA/termination site of the gene. Importantly, expression of RNA Pol II or DHX9 in these cells was unaffected by knockdown of SFPQ (Supplementary Fig. 5c). These data, together with our DRIP experiments, establish that in the presence of SFPQ and SF3B3, DHX9 dissociates from RNA Pol II during transcription elongation. In the absence of SFPQ or SF3B3, DHX9 and RNA Pol II remain closely associated throughout transcription and this correlates with the formation of RNA–DNA hybrids across the entire length of the *Β-actin* coding sequence.

### Tethering of RNA Pol II to chromatin by RNA–DNA hybrid

Although defects in pre-mRNA processing leads to the generation of extensive tracts of RNA–DNA hybrids, recent evidence suggests that R-loops, by themselves, do not necessarily cause genomic instability^46^. Furthermore, while some R-loops elicit a pathological response in cells, others do not, suggesting that only a subset of R-loops are harmful to cells. The reason for this is not clear.

Our experiments revealed that knockdown of SFPQ not only promoted the formation of R-loops across the entire *Β-actin* coding sequence but also caused RNA Pol II to accumulate at the polyA site of the gene. We speculated that the formation of an extended region of RNA–DNA hybrid might impede the dissociation of RNA Pol II from DNA upon termination. This might also increase the potential for TRC and lead to replication fork collapse and increased DNA breaks.

**Fig. 9:**
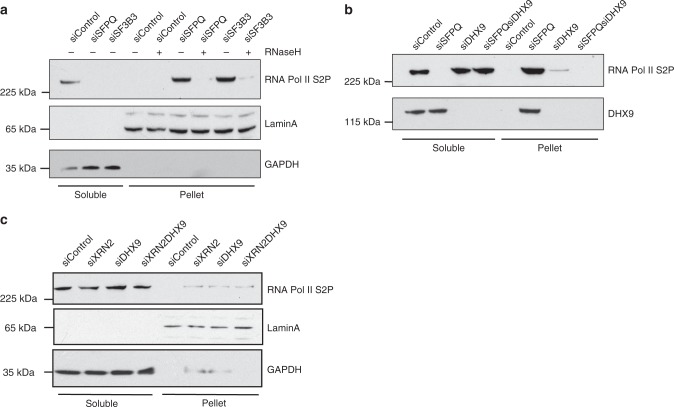
DNA–RNA hybrid traps RNA Polymerase II on chromatin. **a** Depletion of SFPQ and SF3B3 causes retention of RNA Pol II on chromatin that is released by treatment with RNAseH1. Western blot of soluble (cytoplasmic) and insoluble (pellet) fractions of nuclear extracts prepared from HeLa cells that were knocked down with siRNAs against the indicated genes. Blots were also probed for GAPDH and LaminA to validate cytoplasmic and chromatin pellet fractions, respectively. **b** As in **a** showing that knockdown of DHX9 in cells depleted of SFPQ reverses the retention of RNA Pol II S2P on chromatin. **c** RNA Pol II (S2P) is not retained in the insoluble chromatin pellet from cells knocked down for XRN2

To test this idea, we prepared nuclear extracts from wild-type cells and from cells depleted for SFPQ and SF3B3 and examined the presence of RNA Pol II S2P and DHX9 in the soluble nuclear and insoluble chromatin fractions. Western blot confirmed that in extracts prepared from wild-type cells, RNA Pol II S2P and DHX9 were present in the soluble nuclear fraction (Fig. 9a). However, upon depletion of either SFPQ or SF3B3 (Supplementary Fig. 5d), RNA Pol II S2P and DHX9 were greatly enriched in the insoluble pellet, indicating their retention on chromatin (Fig. 9a). Moreover, RNA Pol II was released from chromatin by treating pellets with RNaseH1, suggesting that RNA Pol II was tethered to chromatin by RNA–DNA hybrid (Fig. 9a). Consistent with this hypothesis, knockdown of DHX9 in SFPQ-depleted cells to suppress R-loops prevented the retention of RNA Pol II on chromatin (Fig. 9b).

Interestingly, R-loops formed upon knockdown of XRN2, which do not impair DNA replication, did not cause retention of RNA Pol II on chromatin (Fig. 9c). We suspected, therefore, that it might be the tethered RNA Pol II, rather than RNA–DNA hybrid per se, that blocks DNA synthesis in cells, leading to pathological replication stress and chromosome instability.

We inferred that R-loops formed as a consequence of impaired RNA splicing may involve extensive regions of RNA–DNA hybrids that cause RNA Pol II to be tethered to chromatin where it can block replication and cause genomic instability. R-loops, such as those formed in cells that are depleted of XRN2, which may involve less extensive regions of RNA–DNA hybrid, do not cause retention of RNA Pol II on chromatin and might not lead to the pathological inhibition of DNA replication. Therefore, although pathological and non-pathological R-loops can be formed through a common mechanism involving DHX9, their impact on DNA replication and on genomic instability is probably context-dependent and might also be influenced by other factors.

## Discussion

Although R-loops are required for normal physiological processes, very little is known about how and why they are generated. The demonstration by others that proteins involved in RNA splicing and biogenesis of RNP can suppress R-loop formation established an important link between the generation of R-loops and co-transcriptional processing of pre-mRNA^3^. Our work has now identified DHX9 as a key protein that is required for the formation of, at least, some R-loops and revealed how components of the splicing machinery prevent R-loops from forming by modulating the activity of DHX9 during transcription.

We have shown that DHX9 is required for the formation of R-loops in cells that are defective in splicing factors SF3B3 and SFPQ as well as those treated with the spliceosome inhibitor Pla-B. In the absence of DHX9, the generation of R-loops in these cells is almost entirely suppressed, restoring DNA synthesis and cell growth. This confirms the potential of some R-loops to cause profound replication stress and, more importantly, establishes DHX9 as an important driver of R-loop formation and genomic instability in cells.

In addition to the R-loops that arise as a consequence of perturbed RNA processing, non-pathological R-loops are found throughout the genomes of normal cells^6,8^. Although, we did not address the impact of DHX9 on R-loops globally, we found that the low level of RNA–DNA hybrids that form at the *Β-actin* locus in unperturbed cells are also suppressed by knockdown of DHX9. Furthermore, we demonstrated that R-loops generated in centromeric DNA, which regulate segregation of chromosomes during mitosis, are also dependent on DHX9 for their formation. Together these data strongly argue that, at least some, pathological and non-pathological R-loops are formed through a common mechanism involving DHX9.

Interestingly, DHX9 was recently identified as a component of a RNA–DNA hybrid interactome and shown to suppress R-loops at transcription termination regions and also prevent R-loop associated-DNA damage in cells treated with camptothecin (CPT)^47^. Paradoxically, this study, like our own, reported that siRNA-mediated knockdown of DHX9 caused a global reduction in RNA–DNA hybrids when measured by immunofluorescence and slot blot^47^, supporting our conclusion that DHX9 promotes rather than prevents the formation of R-loops. The reasons for this difference are not clear but it is possible that R-loops induced upon treatment with CPT are generated through a different mechanism to those formed as a consequence of impaired RNA processing.

Although DHX9 unwinds RNA–DNA hybrids in vitro^38^, suggesting that it could function in the removal of R-loops, we have established that, in cells, DHX9 is required for the formation of some R-loops. Since DHX9 also unwinds RNA secondary structures in vitro, including stable guanine quadruplexes, we suggest that it is its ability of DHX9 to unwind RNA that best explains its role in R-loop formation. This is supported by several pieces of evidence. Firstly, DHX9 resolves RNA secondary structures in cells to prevent Alu-mediated backsplicing^36^. Secondly, R-loop formation requires a free RNA end to promote invasion and pairing of the nascent RNA with its complementary duplex DNA^43^. This might be facilitated by DHX9-mediated unwinding of RNA secondary structures to help assemble RNA processing proteins on the nascent RNA strand. Thirdly, the role of splicing factors and other RNA-binding proteins in preventing R-loop formation is most easily explained if they bind to and stabilize the nascent RNA strand, minimizing its potential to invade DNA duplex. This last point is supported by our experimental evidence showing that DHX9 is required for the binding of splicing factors to nascent RNA as part of the elongating transcription complex.

**Fig. 10:**
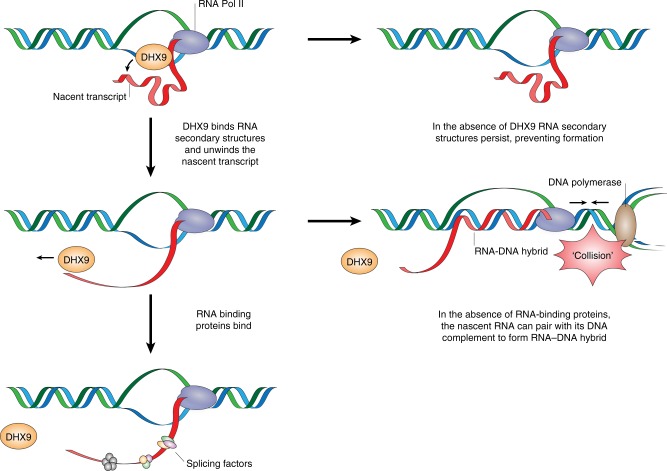
DHX9 promotes the generation of R-loops. Model showing how DHX9 promotes the formation of R-loops by unwinding the nascent RNA to generate the free RNA end, which is required for the invasion of duplex DNA and generation of DNA–RNA hybrid. RNA-binding proteins prevent R-loop formation by binding to the nascent RNA inhibiting its ability to pair with its complementary DNA template. In the absence of RNA-binding proteins, the free RNA end that is generated by DHX9 is available for R-loop formation. This may lead to RNA Pol II becoming trapped on chromatin where it can pose a barrier to DNA replication and increases the likelihood of transcription–replication conflicts. In the absence of DHX9, the formation of secondary structures in the nascent RNA prevent it from invading the DNA duplex to form R-loops

We have also established that the formation of R-loops caused by impaired splicing correlates with the prolonged association of DHX9 with RNA Pol II during transcription. Whereas in normal cells, DHX9 associates with RNA polymerase II in the early phases of transcription, it is absent from RNA Pol II at later stages of elongation. Importantly, the dissociation of DHX9 from RNA Pol II is dependent on the availability of the splicing factors SF3B3 and SFPQ. We hypothesize that these proteins stabilize the unwound nascent RNA strand, preventing it from forming further secondary structures. Since DHX9 does not bind single-stranded RNA^41^, this would alleviate the requirement for DHX9 as transcription progresses. Conversely, in the absence of splicing factors, DHX9 remains associated with the transcription complex where it binds to RNA secondary structures that form in the nascent strand and unwinds them during transcription elongation. The disadvantage of this activity is that, in the absence of splicing factors or components of RNP, the free “unstructured” RNA end generated by DHX9 is available to invade DNA duplex where it can form RNA–DNA hybrid and displaced ssDNA. As transcription progresses, RNA–DNA strand exchange might extend the region of RNA–DNA hybrid to generate an R-loop along an entire coding region as we observed at the *Β-actin* locus. This model, described in Fig. 10, raises the intriguing possibility that it is the impact of splicing defects on transcription, rather than a failure in RNA splicing per se, that contributes to the generation of R-loops^17^. Interestingly, a recent study using a conditional deletion of murine SFPQ proposed that SFPQ, together with CDK9, facilitates transcriptional elongation as well as activating RNA processing and stabilizing pre-mRNA^33^.

In another study, mutations in yeast histones were shown to promote the formation of R-loops without inducing DNA damage, indicating that R-loops, per se, do not cause genome instability^46^. We found that depletion of XRN2 in human cells also induces R-loops but does not cause replication stress. This raised the question, why do some R-loops cause replication stress and genomic instability and others do not? Our data suggest that R-loop formation can cause RNA polymerase to become tethered to chromatin through RNA–DNA hybrid, where it has the potential to block DNA replication. In contrast, R-loops generated upon depletion of XRN2 do not trap RNA Polymerase II on chromatin and these cells do not exhibit replication stress. This supports a model in which it is the retention of RNA Polymerase on chromatin and the concomitant increased transcription–replication collisions that are responsible for pathological replication stress, rather than the generation of R-loops per se.

It is important to note that DHX9 contributes to the processing of genes transcribed by RNA Pol II^48^. Since R-loops can also be generated during transcription by RNA polymerases I and III^6^, there must be other mechanisms through which R-loops form. It will be interesting to determine whether R-loops generated during transcription by these by other polymerases also require the activity of helicases, equivalent to DHX9, that unwind nascent RNA.

In summary, our work identifies DHX9 as a pivotal factor in the formation of R-loops in cells with perturbed RNA splicing. Our data also provide an explanation for the role of splicing factors in the suppression of R-loops, by facilitating the dissociation of DHX9 from RNA Pol II. In the absence of these stabilizing factors, the generation of R-loops causes trapping of RNA Pol II on chromatin creating a physical block to DNA replication and increasing genome instability. Our work reveals detailed new insights into the important interplay between transcription, mRNA splicing, and DNA replication that might be relevant to those neurodegenerative diseases and cancers that are impaired in processing of pre-mRNAs.

## Methods

### Cell culture

HeLa and U2OS cell lines were cultured in Dulbecco’s modified Eagle’s medium supplemented with 10% fetal bovine serum and appropriate antibiotics and grown at 37 °C and 5% CO_2_. All cell lines were obtained from ATCC.

### Antibodies, chemicals, and cell-cycle reagents

Primary antibodies used in this study: mouse anti-RPA32 (RPA2 Ab #2; Calbiochem) used at a dilution of 1:500 for immunofluorescence experiments (IF) and 1:1000 dilution in western blot (WB); rabbit polyclonal anti-SFPQ (Bethyl Laboratories, A301–320A, IF 1:500, IP 4 μg/ml, and WB 1:1000 dilution), mouse monoclonal anti-SFPQ antibody (Abcam, ab11825, IF 1:500 and IP 3.5 μg/ml dilution), mouse monoclonal anti-GAPDH antibody (GeneTex GT239, WB 1:1000 dilution), rabbit polyclonal anti-RNA polymerase II antibody phospho-ser2 (Abcam, ab5095, IP:2 μg/ml and WB 1:1000 dilution), rabbit Polyclonal anti-RNA polymerase II antibody phospho-ser5 (Abcam, ab52208, IP: 2 μg/ml and WB 1:1000 dilution), rabbit polyclonal anti-Chk1 [p Ser345] (Novus Biologicals, WB 1:1000 dilution), rabbit Polyclonal anti-Phospho RPA32 (S4/S8) Antibody (Bethyl Laboratories, A300-245AWB 1:1000 dilution), rabbit polyclonal anti-RNA polymerase II antibody phospho-ser7 (Abcam, ab126537, IP: 2 μg/ml and WB 1:1000 dilution), rabbit polyclonal anti-SF3B3 antibody (Abcam, ab96683, WB 1:1000 dilution), mouse monoclonal anti-γH2AX (ab26350, Abcam, IF 1:100 dilution and WB 1:500 dilution), rabbit polyclonal anti-53BP1 Antibody (Novus Biologicals, NB100-904 IF 1:500, WB 1:2000, IP 2 mg/ml and NB100-304 IP 2 mg/ml), rabbit polyclonal anti-RNA Helicase A antibody (ab26271, WB 1:1000 dilution and IP 4 mg/ml), rabbit polyclonal anti-XRN2 Antibody (Cambridge Biosciences A301-103A, WB 1:1000 dilution), mouse monoclonal anti-RNase H1 antibody (ab56560, WB 1:1000 dilution) and rabbit polyclonal anti-nucleolin antibody (ab22758, WB 1:1000 dilution), rabbit monoclonal anti-SC35 (SRSF1) antibody (Abcam ab204916, WB 1:2000 dilution). For detecting DNA–RNA hybrid, mouse anti-DNA–RNA Hybrid [S9.6] (Kerafast, ENH001, 1:100 dilution for IF and 1:1000 dilution for slot blot) was used and mouse anti-DNA, single stranded (Merck Millipore, MAB3034 1:1000 dilution for dot blot) to detect total ssDNA. Secondary antibodies: goat anti-mouse Alexa Fluor-488 (Molecular Probes) was used at 1:200 dilution, and goat anti-rabbit alexa fluor 647 (Molecular Probes) was used at 1:200 dilution. EdU incorporation was measured by using the Click-iT EdU Alexa Fluor 647 flow cytometry kit (Life Technologies) according to the manufacturer’s instructions. Hydroxyurea (H8627) and Actinomycin D (A1410) were supplied by Sigma-Aldrich and Pladienolide B (CAS 445493-23-2) was purchased from Santa-Cruz Biotechnology.

### Plasmids

pSFPQ-myc is described in Rosonina et al.^49^. pCMV-DHX9-GFPSPARK is designated pGFP-DHX9 and was purchased from Stratech (Sino-Biologicals). pGFP-DHX9dead contains D511A and E512A mutations made by site-directed mutagenesis of pGFP-DHX9 using Q5 Site Directed Mutagenesis system (New England Biolabs) according to the manufacturer’s instructions.

### SiRNA

The siRNAs were purchased from Dharmacon-ON-TARGETplus. Non-targeting siRNA D-001810-01-05; SFPQ-J-006455-06 J-006455-08 J-006455-09; SFPQ utr DUPLEX STD CTM-64991; SF3B3-J-20085-08; DHX9-J-009950-06 DHX9utr CTM-310164; RNASEH1-J-012595-09; XRN2- J-017622-12

### qPCR primers

Primers used for qPCR analysis of the *Β-actin* locus are described in Kaneko et al.^39^.

Region A: (fwd) cccacctgacaacctctcat, (rev) cccttcttgctgcctgtt

Promoter: (fwd) ctcaatctcgctctcgctct, (rev) ctcgagccataaaaggcaac

Region B: (fwd) caactgggacgacatggagaaa, (rev) gagtcctacggaaaacggcaga

Region C: (fwd) gcgcacagtaggtctgaaca, (rev) agaggcgtacagggatagca

Poly(A): (fwd) tgtacactgacttgagaccagt, (rev) aagcaggaacagagacctgacc

Region D: (fwd) taggcttaggagaggccgcaat, (rev) gtccaggagcctgggtatctcc

Region E: (fwd) gaggcctggactctcaactg, (rev) ggtccttgtccaggtcatct

Region F: (fwd) caaccagatgtgttccgtgt, (rev) ctacacctgcaagaccacca

Region G: (fwd) actgctgacattggtgatgc, (rev) agtaggtggtggcagcagac

Region H: (fwd) tgtctcagaggcatggattg, (rev) ccgctacagtcaccttccag

Primers used for DRIP at the centromeric region of Chromosome 1 are described in Kabeche et al.^2^.

c1 centro (fwd) tcattcccacaaatgcgttg, (rev) tccaacgaatgggaaaggagtc

c1 pericentro (fwd) catcgaatggaaatgaaaggagtc, (rev) accattggatgattgcagtcaa

snrpn (fwd) gccaaatgagtgaggatggt, (rev) tcctctctgcctgactccat

### Cell fractionation

Briefly, 3 × 10^6^ HeLa cells per condition were collected and suspended in 250 µl of buffer A (10 mM HEPES pH 7.8, 10 mM KCl, 1.5 mM MgCl_2_, 0.25 M sucrose, 10% glycerol, 1 mM DTT, 0.1% Triton X-100, protease and phosphatase inhibitors) and incubated for 5 min on ice. The soluble cytoplasmic fraction (S1) was separated from the nuclei (P2) by centrifugation for 4 min at 2000 × *g* at 4 °C. The nuclear fraction P2 was washed twice with 500 µl buffer A and suspended in 200 µl buffer B (3 mM EDTA, 0.2 mM EGTA, 1 mM DTT, phosphatase, and protease inhibitors) and incubated at 4 °C for 30 min. The insoluble chromatin fraction (P3) was separated from nuclear soluble proteins (S3) by centrifugation for 4 min at 1700 × *g* at 4 °C. S1 was cleared from insoluble proteins by centrifugation at 16,000 × *g* in a benchtop micro-centrifuge for 30 min at 4 °C and the supernatant (S2) was kept for analysis. Cell fractions were subsequently analyzed by the Novex transfer system (Invitrogen) and western blotting.

### Immunoprecipitation

Prior to immunoprecipitation, primary antibody was incubated with Dynabeads protein G beads (Novex, Invitrogen) for 2–4 h at 4 °C. Nuclei were prepared as described above. For immunoprecipitation of DHX9, nuclei were lysed using Lysis Buffer (150 mM NaCl, 0.5% Triton X-100, 50 mM Tris-HCl pH 7.5, 1 mM EDTA, 5% glycerol, and 0.1% SDS) supplemented with protease and phosphatase inhibitor cocktails (Thermoscientific). Extracts were passed through 23G needle for 10 min, and incubated with rocking at 4 °C for 30 min. Extracts were centrifuged at 16,000 × g for 15 min in a microfuge and the supernatant was collected. For immunoprecipitation of RNA Pol II (phospho-ser-2), nuclei were lysed in Nuclear Lysis buffer (100 mM Tris pH 7.5, 350 mM NaCl, 1% Triton X-100 and 10% glycerol). DNA in the extract was digested overnight by the addition of 50 units each of *Bam*HI, *Nco*I, *Pvu*II, *Apa*LI, *Nhe*I, *Xba*I, *Xmn*I, and *Dra*I. The supernatant was cleared by centrifugation at 16,000 × *g* for 15 min and extracts were left untreated or treated with RNaseA (10 mg/ml).

Extract was added to the antibody-Dynabead complex and then incubated with rotation for 4 h to overnight at 4 °C. Immunocomplexes were separated using a magnet, washed three times in lysis buffer, boiled in sample buffer, and loaded on a 4–12% Bis-Tris polyacrylamide gel (Invitrogen). Proteins were transferred to PVDF membrane using a Novex transfer system (Invitrogen) and immunoblotted using the indicated antibodies.

### Immunofluorescence microscopy

For EdU incorporation, U2OS cells were seeded onto coverslips and transfected with the indicated siRNA and grown up to 96 h. At the indicated times 10 μm EdU was added and incubation continued for a further 1 h before cells were harvested. Coverslips containing fixed cells were washed with phosphate-buffered saline (PBS) and stained using the Click-iT EdU Alexa Fluor 647 Flow cytometry kit (Life Technologies) according to the manufacturer’s instructions. For detection of nuclear foci, cells were pre-extracted for 5 min on ice in 25 mM Hepes pH 7.4, 50 mM NaCl, 1 mM EDTA, 3 mM MgCl_2_, 300 mM sucrose, and 0.5% Triton X-100, after which they were fixed using 4% formaldehyde (w/v) in PBS for 15 min. Cells were then washed three times in PBS and permeabilized for 10 min in 0.5% Triton X-100/PBS. Three additional PBS washes were performed, after which cells were blocked using 1–3% bovine serum albumin (BSA)/PBS for 30 min. Cells were incubated with primary antibody (as indicated above), followed by the addition of Alexa Fluor-488 or -594 conjugated secondary antibodies (1:1000). To visualize nuclei, cells were also stained with 0.5 μg/ml DAPI (Molecular Probes). Slides were mounted using Prolong gold anti-fade reagent (Invitrogen) and images were acquired using a Deltavision DV4 wide-field deconvolution microscope with a ×100 objective.

To detect DNA–RNA hybrid, cells were stained with S9.6 antibody as described^27^. Cells were co-stained with antibody against nucleolin, which was used to mask nucleolar regions for quantification of S9.6 staining. The nuclear S9.6 signal was determined subtracting the S9.6 staining intensity of the region co-stained with nucleolin from the total nuclear S9.6 stain. Images were analyzed using a DeltaVision microscope and quantified using image J software.

### Cell growth assay

U2OS cells were transfected with siRNA for 48 h and 50,000 cells were seeded into a six-well plate (time 0). At the indicated times, cells were recovered from the monolayer using trypsin and counted using the Casey Cell Counter. Growth curves were plotted using data from three independent biological replicates.

### Cell-cycle analysis

Cells were grown for 1 h in medium supplemented with 10 μm BrdU. Cells were fixed using ethanol and the DNA was denatured using 1 ml of 2 N HCl/Triton X-100 and then neutralized with 500 μl 0.1 M Na_2_B_4_O_7_. Cells were blocked using PBS/1% BSA/0.5% Tween-20 and incubated with mouse anti-BrdU primary antibody (clone BU20A, Dako) followed by goat anti-mouse FITC-conjugated secondary antibody (DAKO). Cells were suspended in 1 ml of PBS containing 5 μg/ml propidium iodide and 5 μl RNAse (25 mg/ml), incubated at room temperature for 30 min, and analyzed by FACS. Cell-cycle distribution was measured by flow cytometry in a Fortessa flow cytometer (Becton Dickinson) and analyzed using FlowJo software (FlowJo).

### Annexin-V assay

Apoptotic cell death was analyzed using Annexin-V kit (ebiosciences) as described by the manufacturer and quantified using a NucleoCounter NC-3000 system (Chemomotek).

### DNA–RNA immunoprecipitation

DRIP was performed using HeLa cells transfected with the indicated siRNAs. Briefly, each immunoprecipitation used 10 × 10^6^ cells that were lysed in cell lysis buffer (1 M KCL, 100 mM HEPES pH 7.8 and 1% Triton X-100) for 5–10 min on ice. Cells were washed with PBS, collected by centrifugation and the collected nuclei lysed in nuclei lysis buffer (1 M Tris pH 8, 0.1 M EDTA and 10% SDS). Proteinase K (10 mg/ml) was added and nuclei pellet were incubated at 37 °C overnight. Nucleic acid was precipitated using ethanol and digested overnight by the addition of 50 units each of *Bam*HI, *Nco*I, *Pvu*II, *Apa*LI, *Nhe*I, *Xba*I, *Xmn*I, and *Dra*I. Digested nucleic acid was treated with RNaseA (10 mg/ml) and 5 M NaCl for 2 h at 37 °C to remove contaminating ssRNA. DNA:RNA hybrids were precipitated using glycogen and isopropanol. Nucleic acid was purified with phenol–chloroform and precipitated with ethanol. The DNA was rehydrated in 250 μl water and quantified with a Nanodrop spectrophotometer. Fifty microliters was stored as the “input” sample. Ten micrograms of nucleic acid was treated with 25 U RNaseH1 to digest DNA–RNA hybrid and serve as a negative control. Five micrograms of RNAseH1 treated and untreated nucleic acid was immunoprecipitated using 10 μg S9.6 antibody and 50 μl with Dynabeads Protein G and made up to 500 μl with binding buffer (1 M Na-phosphate pH 7, 5 M NaCl, and 10% Triton X-100) and incubated overnight at 4 °C. IPs were then washed several times with wash buffer (1 M Tris pH 8, 500 mM EDTA, 10% SDS, 10% Triton X-100, 5 M NaCl). Nucleic acid were eluted with 100 mM NaHCO_3_ and 1% SDS along with addition of 3 μl RNaseA for 2 h at 65 °C followed by incubation with Proteinase K (10 mg/ml). Proteins were removed from the sample by phenol–chloroform extraction and ethanol precipitation. Control IP was performed using 10 μg of anti-mouse IgG antibody. Quantitative PCR was performed using Fast SYBR™ Green Master Mix (Applied Biosystems) with the indicated primers and samples were amplified using an Applied Biosystems 7500 Real-Time PCR System. The data were expressed as fold enrichment of each primer region in the experimental sample over the negative control. The mean and s.e.m. was plotted from three independent experiments and statistical analysis was performed using one-way ANOVA. The list of primers are provided above.

### Slot blot

Preparation of nucleic acids was as described for DRIP with a few modifications. Briefly, after overnight digestion with restriction enzymes half of the nucleic acid sample was treated with RnaseH1 (20 U) every 3 h. The other half of the sample was treated with denaturation buffer (0.5 N NaOH and 1.5 M NaCl) and then neutralized with 1 M NaCl and 0.5 M Tris-HCl pH 7.0. In all, 0.5, 1, and 2 μg aliquots of nucleic acids were loaded onto a nylon membrane using a Biorad slot blot manifold and then crosslinked using a Uvitech (Cambridge) Stratalinker (0.12 J/m^2^). Membranes were blocked in 5% Milk with PBST and probed overnight at 4 °C with S9.6 antibody (diluted 1:1000) and anti-mouse ssDNA antibody (1:5000) and then probed with goat anti-mouse antibody secondary antibody. The membrane was washed three times with PBST and exposed to X-ray film. The image is normalized to the control sample (set to 1) and quantified using Image J software.

### Chromatin immunoprecipitation

1–5 × 10^7^ HeLa cells were transfected with siRNA for 48–72 h, washed, and crosslinked with 0.5–1% formaldehyde at room temperature for 10 min for RNA Pol II and 3% formaldehyde at room temperature for DHX9 immunoprecipitation. One hundred and twenty-five millimolar glycine was added directly to cells in 15 cm dishes and incubated for another 5 min with shaking. Cells were washed and harvested in cold PBS. Cells were collected by centrifugation at 1000 × *g* for 5 min at 4 °C and suspended in lysis buffer (50 mM HEPES-KOH pH 7.5, 140 mM NaCl, 1 mM EDTA pH 8, 1% Triton X-100, 0.1% sodium deoxycholate 0.1% SDS and Protease Inhibitors cocktail). Lysate was digested overnight with the addition of 50 units each of *Bam*HI, *Nco*I, *Pvu*II, *Apa*LI, *Nhe*I, *Xba*I, *Xmn*I, and *Dra*I. The lysate was centrifuged at 15,700 rcf for 15 min in a benchtop microfuge and the supernatant collected. Twenty-five microliters of Dynabeads coupled to 4 μg anti-DHX9 antibody was added to 500 μl supernatant and incubated with rotation at 4 °C overnight. Antibody was omitted from one sample as a control. Immunocomplexes were separated using a magnet, washed three times in lysis buffer, and DNA eluted in 200 mM glycine for 5 min and then boiled for 10 min. Immunocomplexes were incubated with 30 units RNase A for 30 min at 37 °C followed by proteinase K for 45 min at 55 °C. DNA was purified with phenol–chloroform and quantified using a Nanodrop spectrophotometer (Thermo-Fisher). Quantitative PCR was performed using standard protocols in an ABI PRISM 7900 (Applied Biosystems) using Fast SYBR Green Master Mix (Applied Biosystems). Analysis was carried out using the delta Ct method (fold enrichment). The mean and s.e.m. was plotted from three independent experiments and statistical significance determined using one-way Anova.

### Data processing and statistical analysis

Values are shown with the standard deviation from three independent experiments unless indicated otherwise. Data were analyzed and, where appropriate, the significance of the differences between the mean values was determined using two-tailed Students *t*-test (*p ≤ 0.05), Mann–Whitney unpaired *t*-test (**p ≤ 0.01, ****p ≤ 0.0001) or one-way Anova test as indicated. All statistics were performed using Prism v6 (GraphPad Software).

## Electronic supplementary material

Supplementary Information

## Data Availability

The data that support the findings of this study are available from the corresponding author upon reasonable request.
