# Supplementary material for: DHX9 helicase promotes R-loop formation in cells with impaired RNA splicing
Source: Nat Commun. 2018 Oct 19;9:4346. doi: 10.1038/s41467-018-06677-1 (PMC6195550; doi:10.1038/s41467-018-06677-1)
Supplement: Supplementary file 1 — Supplementary Information [file 41467_2018_6677_MOESM1_ESM.pdf]

a

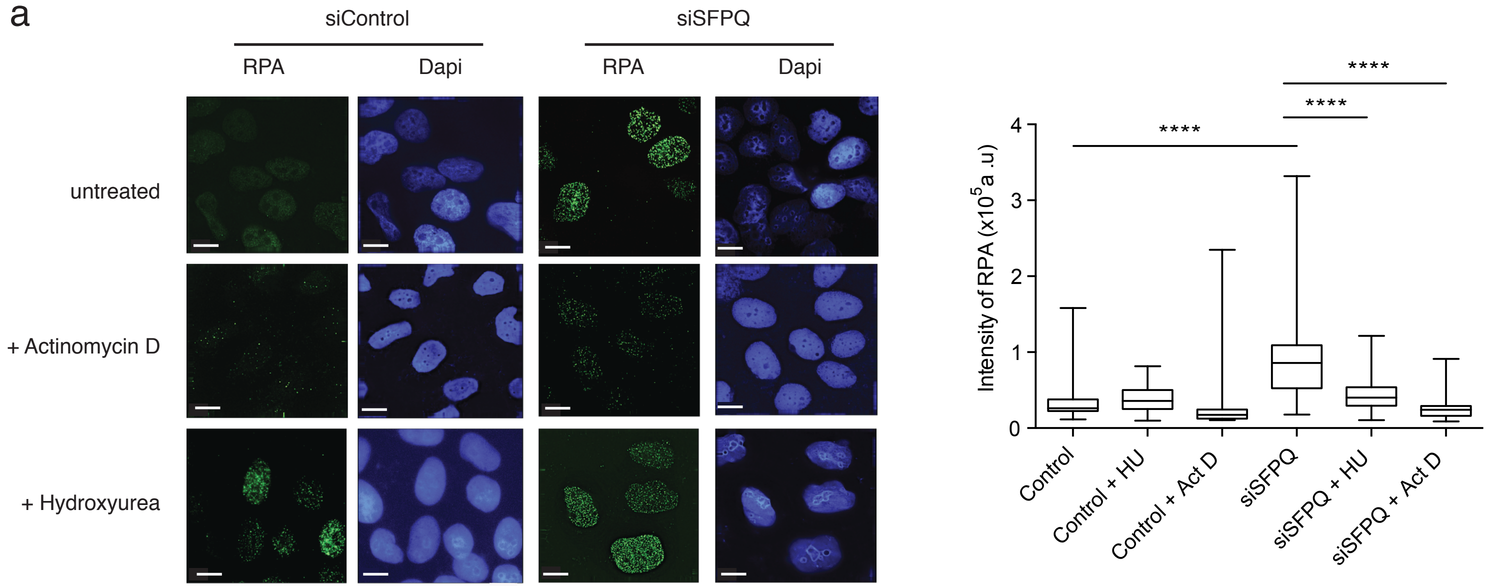

b

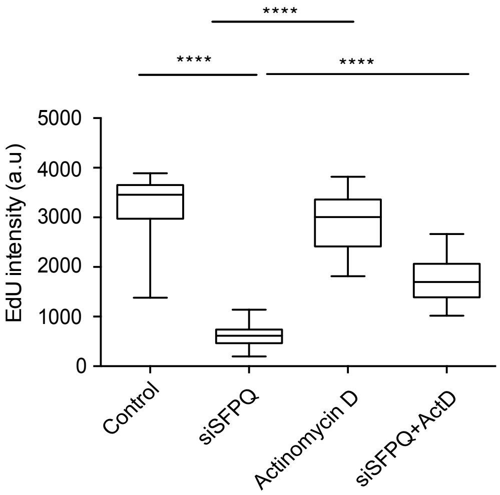

Supplementary Figure 1. (a) Microscope images showing cells stained with antibody against RPA. Cells were knocked down with siControl or siSFPQ9 and treated with Actinomycin D (0.5 μg/ml, 2 hours) or with hydroxyurea (2mM, 4 hours) as indicated. Immunofluorescence intensity per nucleus from >100 cells is presented graphically as a box and whiskers plot. (b) Graph showing restoration of replication in siSFPQ knockdown cells after inhibition of transcription with Actinomycin D. EdU fluorescence intensity from >100 cells is presented graphically in arbitrary units (a.u.). Statistical significance for box and whiskers graphs was determined using Mann Whitney test (****p<0.0001).

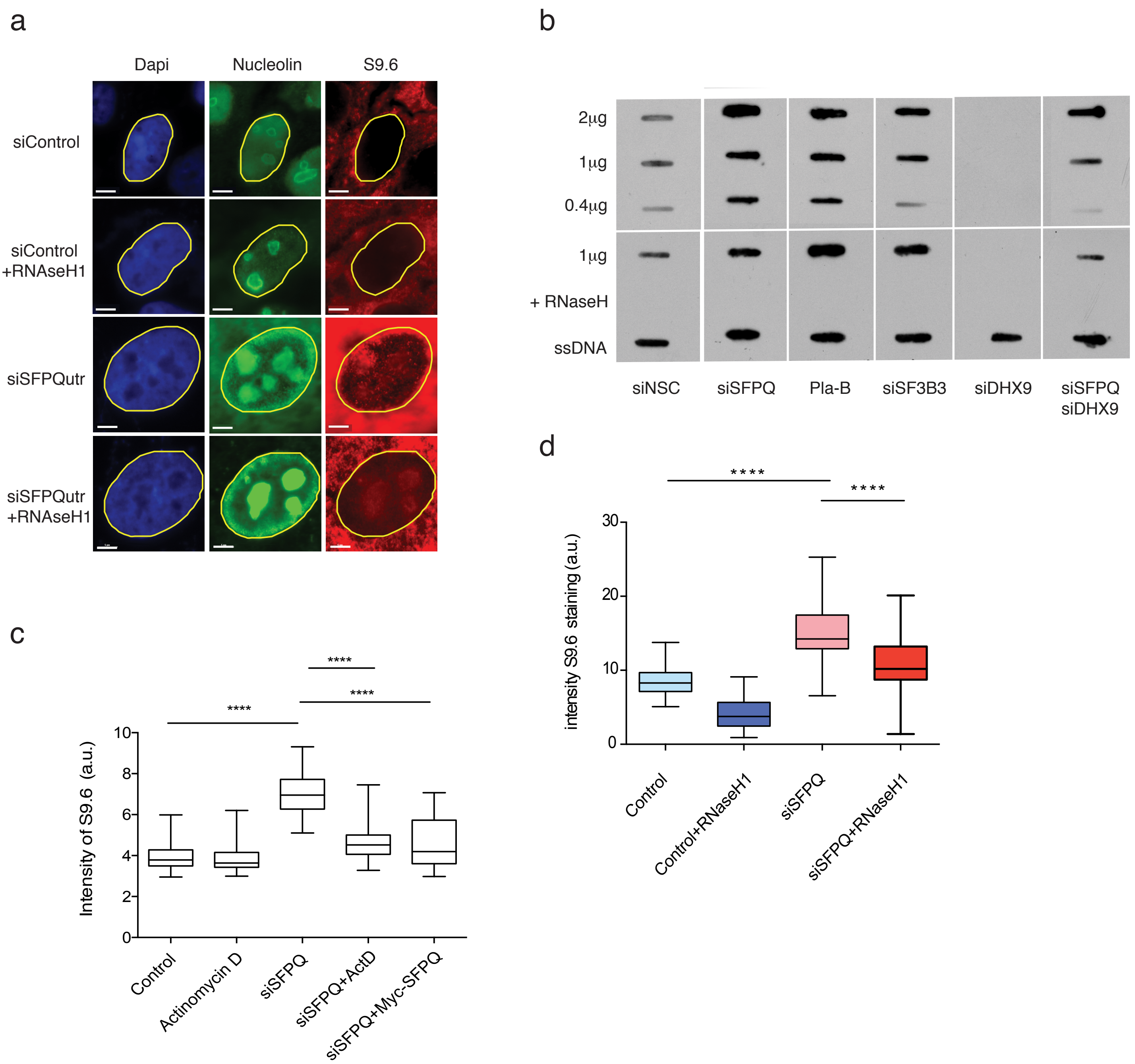

Supplementary Figure. 2. (c) Representative images showing immunostaining for RNA-DNA hybrid using S9.6 antibody (red) and for nucleolin (green). Hela cells were transfected with siControl or siSFPQutr as indicated. Where indicated, cells were treated with RNAseH1. Nuclei were visualised using DAPI stain. Scale bars represents 5 µm. (b) Slot blot hybridization of RNA-DNA hybrid immunoprecipitated from HeLa cells knocked down with the indicated siRNAs. Where indicated, cells were treated with Pladienolide B (5 µM) for 2 hours. Each sample corresponds to 0.4 µg, 1 µg or 2 µg nucleic acid spotted onto nitrocellulose and stained with S9.6 antibody. Identical samples were treated with RNaseH1 treatment (5U RNaseH1 for 6 hours) as indicated. Staining for ssDNA is shown as a loading control (c) Graph showing restoration of replication in siSFPQ knockdown cells expressing siRNA resistant myc-SFPQ. Quantification of EdU fluorescence intensity from >100 cells is presented graphically in arbitrary units (a.u.). Statistical significance for box and whiskers graphs was determined using Mann Whitney test (****p<0.0001). (d) Knockdown of SFPQ (siSFPQ8) causes increased production of RNA-DNA hybrid. The fluorescence intensity of S9.6 antibody staining was measured for >100 cells and is represented in arbitrary units (au). Where indicated, cells were treated with RNAseH1 to degrade RNA-DNA hybrid.

DHX9 peptides detected in a SFPQ pull-down experiment

Unique	Pepetide	Score	Mass	Mass error (ppm)	m/z	Modification
TRUE	R.AAEC(+57.02)NIVVTQPR.R	30.48	1356.682	-0.3	679.3481	Carbamidomethylation
TRUE	R.ISAVSVAER.V	29.41	930.5134	-0.1	466.2639	
TRUE	K.LAAQSC(+57.02)ALSLVR.Q	25.68	1287.697	-1.5	644.8547	Carbamidomethylation
TRUE	R.DVVQAYPEVR.I	24.25	1174.598	-1.2	588.3057	

SFPQ peptides detected in a DHX9 pull-down experiment

Unique	Pepetide	Score	Mass	Mass error (ppm)	m/z	Modification
TRUE	R.FGQGGAGPVGGQGPR.G	50.53	1340.658	0.2	671.3366	
TRUE	K.YGEPGEVFINK.G	50.31	1251.614	-0.2	626.8139	
TRUE	R.FATHAAALSVR.N	46.64	1142.62	0.4	381.8806	
TRUE	R.AVVIVDDR.G	46.52	885.4919	-0.6	443.753	
TRUE	R.FATH(+57.02)AAALSVR.N	43.85	1199.641	-0.6	600.8275	Carbamidomethylation
TRUE	K.Y(+57.02)GEPGEVFINK.G	43.3	1308.635	-0.2	655.3246	Carbamidomethylation
TRUE	K.GIVEFASKPAAR.K	39.1	1244.688	-1	623.3505	
TRUE	K.YGEPGE(+57.02)VFINK.G	36.6	1308.635	-2.4	655.3232	Carbamidomethylation
TRUE	R.F(+57.02)ATHAAALSVR.N	36.28	1199.641	-0.7	600.8274	Carbamidomethylation
TRUE	R.S(+57.02)RGGGGGGFHR.R	34.06	1100.522	7.6	367.8508	Carbamidomethylation
TRUE	R.FGQGGAGPVGGQGPRG.M	33.49	1397.68	-0.2	699.8471	
TRUE	K.GIVEFASK(+57.02)PAAR.K	32.03	1301.709	0.4	651.8621	Carbamidomethylation
TRUE	R.A(+57.02)VVIVDDR.G	30.6	942.5134	-0.7	472.2636	
TRUE	K.ANLSLLR.R	26.16	785.4759	0.3	393.7453	
TRUE	K.AELDDTPM(+15.99)R.G	25.02	1062.465	-1.6	532.239	Oxidation (M)
TRUE	R.ALAEIAK.A	24.99	714.4276	0.9	358.2214	
TRUE	R.AVVIVD(+57.02)DRGR.S	24.04	1155.636	-1.3	578.8245	Carbamidomethylation
TRUE	R.LFVGNLPADITEDEFKR.L	22.99	1963.005	-0.3	655.3421	

Supplementary Figure 3. Lists of peptides identified by mass spectrometry from immunoprecipitation of DHX9 and SFPQ, confirming that DHX9 and SFPQ interact. The pull-down proteins were resolved on a SDS-PAGE gel before subjecting to in-gel digestion. The database search was performed on PEAKS search engine against an Uniprot human database (version 2018-05-28). The false discovery rate (protein level) was set at 1%.

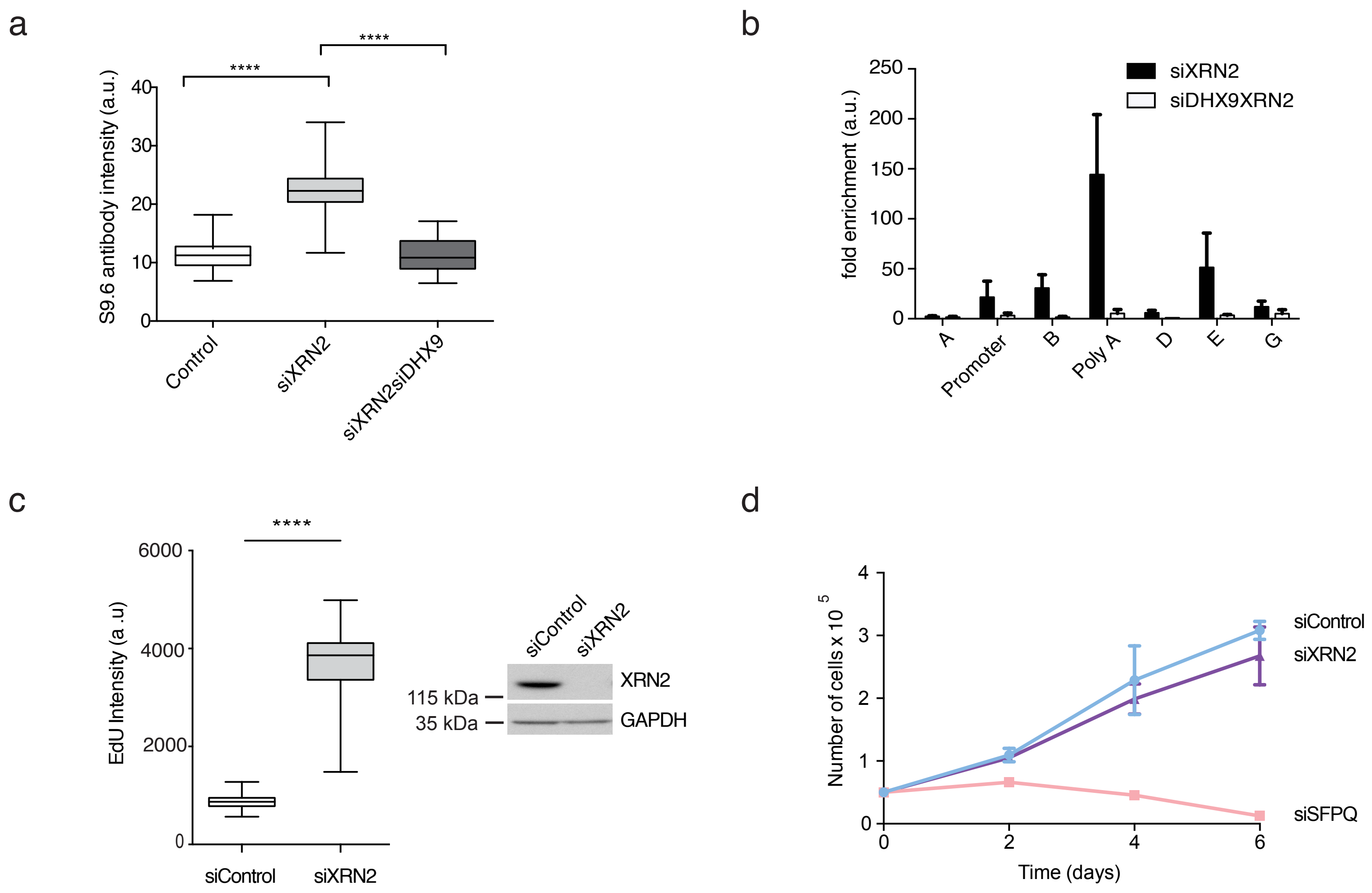

Supplementary Figure 4. (a) Knockdown of XRN2 induces formation of R-loops which are suppressed by depletion of DHX9. (b) DNA-RNA immunoprecipitation (DRIP) at the B-actin locus. RNA-DNA hybrid was immunoprecipitated using S9.6 antibody from U2OS cells knocked with the indicated siRNAs. Quantitative PCR of IP samples was performed using the primers indicated (see Fig. 6) and data are represented as fold enrichment over control samples for the indicated regions of the B-actin locus. Data is an average of three independent experiments. (c) DNA synthesis is enhanced in cells knocked down for XRN2. Quantifiation of EdU fluorescence from >50 cells is presented in arbitrary units (a.u.). Statistical significance was determined using Mann Whitney test (**** P<0.0001). Western blot confirming knockdown of XRN2 in U2OS is shown (d) Proliferation of U2OS cells transfected with siRNAs targeting SFPQ (siSFPQ8), siXRN2 or a non-specific sequence (siControl). Cell number was measured 48 hours after transfection with siRNA (time =0) and again every 2 days. Data represent mean of three independent replicates ± s.d.

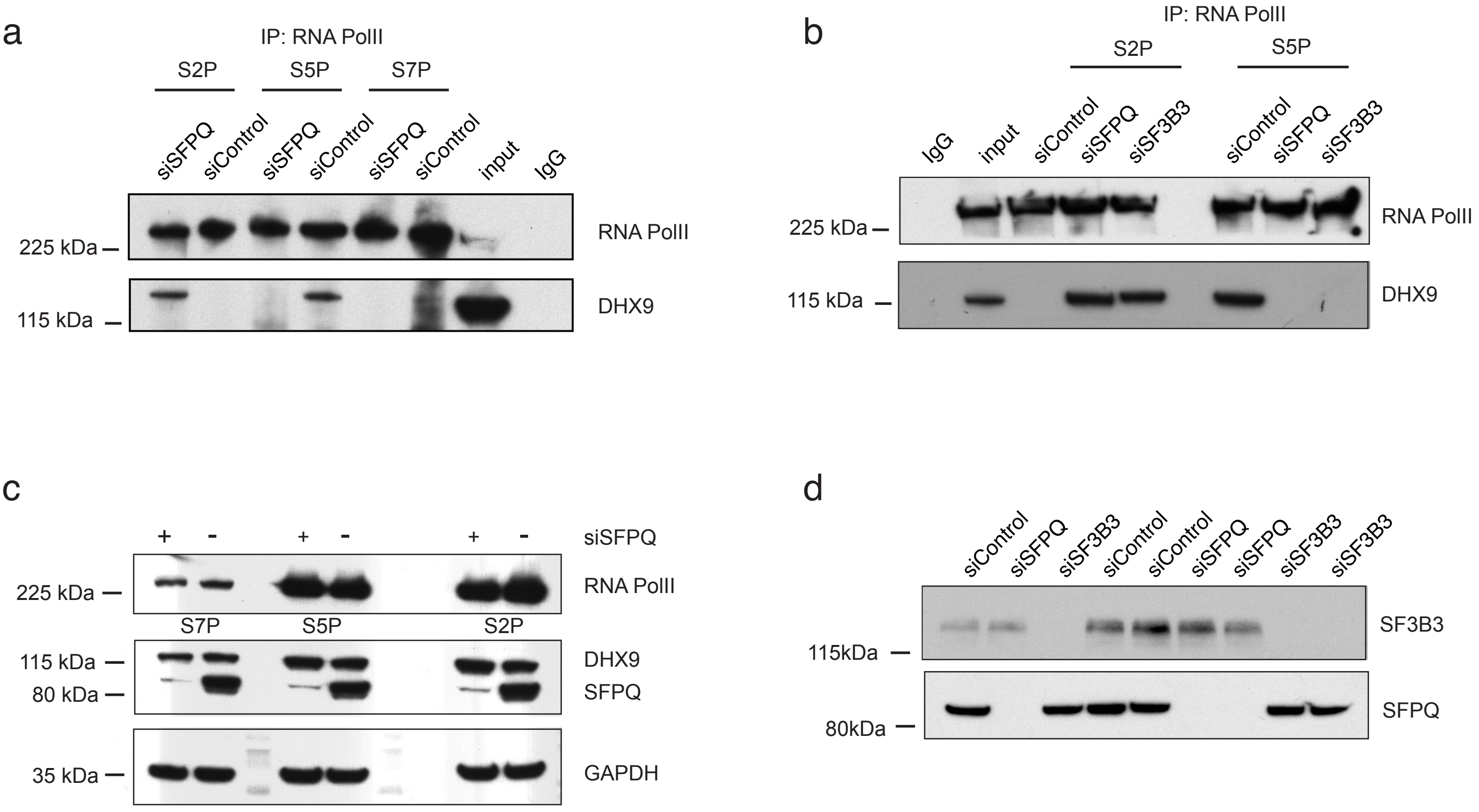

Supplementary Figure 5. (a) DHX9 associates with different phosphorylated forms of RNA Pol II in the presence and absence of SFPQ. Western blot of RNA Pol II and DHX9 immunoprecipitated from HeLa cells with antibodies specific for phosphor-serine 2 (S2P), phosphor-serine 5 (S5P) and phosphor-serine 7 (S7P) forms of RNA Pol II. Where indicated cells were transfected with siRNA against a scrambled DNA sequence (siControl) or against SFPQ (siSFPQ8, siSFPQ9). (b) In the absence of SF3B3, DHX9 associates with mainly with the S2P form of RNA PolII. Western blot of RNA Pol II and DHX9 immunoprecipitated from HeLa cells with antibodies specific for S2P and S5P forms of RNA Pol II. (c) Western blot showing that expression of the different modified forms of RNA Pol II and DHX9 were unaffected by knockdown of SFPQ. SFPQ was knocked down for 48 hours and samples analysed by western blot with antibodies against the indicated proteins. (d) Western blot showing knockdown of SFPQ and SF3B3 for experiment described in Fig. 9(a).
